# *ALCAM* (CD166) as a gene expression marker for human mesenchymal stromal cell characterisation

**DOI:** 10.1016/j.gene.2020.100031

**Published:** 2020-03-14

**Authors:** Bas Brinkhof, Bo Zhang, Zhanfeng Cui, Hua Ye, Hui Wang

**Affiliations:** a Institute of Biomedical Engineering, Department of Engineering Science, University of Oxford, Oxford, United Kingdom; b Oxford Suzhou Centre for Advanced Research, Suzhou Industrial Park, Jiangsu 215123, China

**Keywords:** MSC, RNA, Regenerative medicine, Activated-leukocyte cell adhesion molecule, qPCR, Biomarker, MSC, Mesenchymal Stromal Cells, BM, bone marrow, UC, umbilical cord, AD, adipose, DP, Dental Pulp, OS, Osteosarcoma, PL, Placenta, MM, Multiple Myeloma, AF, Amniotic Fluid, ISCT, International Society for Cell and Gene Therapy, CD, cluster of differentiation, (q)PCR, (quantitative) polymerase chain reaction, ALCAM, Activated-Leukocyte Cell Adhesion Molecule, BSG, Basigin, CLIC1, chloride intracellular channel 1, CLIC4, chloride intracellular channel 4, EDIL3, EGF like repeats and discoidin domains 3, ENG, Endoglin, EPHA2, EPH receptor A2, FN1, Fibronectin 1, IGFBP7, insulin like growth factor binding protein 7, ITGA1, integrin subunit alpha 1, LAMP1, lysosomal associated membrane protein 1, LRRC59, leucine rich repeat containing 59, MCAM, melanoma cell adhesion molecule, NECTIN2, nectin cell adhesion molecule 2, NT5E, 5′-nucleotidase ecto, PPIA, peptidylprolyl isomerase A, PUM1, pumilio RNA binding family member 1, TBP, TATA-box binding protein, TFRC, transferrin receptor, THY1, Thy-1 cell surface antigen, TLN1, Talin 1, TMEM47, transmembrane protein 47, YWHAZ, tyrosine 3-monooxygenase/tryptophan 5-monooxygenase activation protein zeta, FACS, Fluorescence Assisted Cell Sorting, RNA-seq, RNA sequencing, cDNA, DNA complementary to RNA, TCF, Tissue Culture Plate, DF, Dermal Fibroblasts, RT, Reverse Transcriptase, Cq, Quantification cycle, SEM, Standard Error of the Mean, MPC, Mesenchymal Progenitor Cell, ER, Endoplasmatic Reticulum, NK, Natural Killer, RM, Regenerative Medicine, TE, Tissue Engineering

## Abstract

**Background:**

Human mesenchymal stromal cells (MSCs) phenotypically share their positive expression of the International Society for Cell and Gene Therapy (ISCT) markers CD73, CD90 and CD105 with fibroblasts. Fibroblasts are often co-isolated as an unwanted by-product from biopsy and they can rapidly overgrow the MSCs in culture. Indeed, many other surface markers have been proposed, though no unique MSC specific marker has been identified yet. Quantitative PCR (qPCR) is a precise, efficient and rapid method for gene expression analysis. To identify a marker suitable for accurate MSC characterisation, qPCR was exploited.

**Methods and results:**

Two commercially obtained bone marrow (BM) derived MSCs and an hTERT immortalised BM-MSC line (MSC-TERT) have been cultured for different days and at different oxygen levels before RNA extraction. Together with RNA samples previous extracted from umbilical cord derived MSCs and MSC-TERT cells cultured in 2D or 3D, this heterogeneous sample set was quantitatively analysed for the expression levels of 18 candidate MSC marker genes. The expression levels in MSCs were compared with the expression levels in fibroblasts to verify the differentiation capability of these genes between MSCs and fibroblasts. None of the ISCT markers could differentiate between fibroblasts and MSCs. A total of six other genes (*ALCAM*, *CLIC1*, *EDIL3*, *EPHA2*, *NECTIN2*, and *TMEM47*) were identified as possible biomarkers for accurate identification of MSCs.

**Conclusion:**

Justified by considerations on expression level, reliability and specificity, Activated-Leukocyte Cell Adhesion Molecule (*ALCAM*) was the best candidate for improving the biomarker set of MSC identification.

## Highlights

• *ALCAM* showed reliable high gene expression levels in hMSCs from different sources and under different culture conditions
• *ALCAM* gene expression can distinguish between several hMSC-lines and fibroblasts
• *EDIL3* and *TMEM47* gene expression can aid in distinguishing between hMSCs and fibroblasts
• *EDIL3* and *TMEM47* and *ALCAM* in particular, could possibly replace *ENG* and *THY1* in improved hMSC characterisation.

## 1 Introduction

Mesenchymal stromal cells (MSCs) are a valuable type of cells in regenerative medicine for their ease of isolation and multipotency. They can be isolated from virtually every organ or tissue in the post-natal body (da Silva Meirelles, 2006) and be differentiated in vitro into several cell types (Caplan, 2017). MSCs are traditionally defined by: 1) the ability to adhere to plastic, 2) tri-lineage differentiation potential, and 3) CD105+, CD90+, CD73+, and CD45−, CD34−, CD14− or CD11b−, CD79α− or CD19− and HLA-DR− in their surface marker expressions. Since the publication of these minimal criteria to define MSCs in 2006 (Dominici et al., 2006), the acronym and the hMSC criteria have been under debate lately (Boregowda et al., 2018; Caplan, 2017; Robey, 2017). This discussion is partially based on the inconsistent or even contradictory research results (Zhang et al., 2015), probably due to a lack of uniformity in nomenclature, no reference cell type and/or the lack of information on the process of generating MSCs (Reger and Prockop, 2014; Viswanathan et al., 2014). Furthermore, fibroblasts, a mature mesenchymal cell type particularly abundant in connective tissues, share phenotypic expression of CD90 (Stern, 1973; Walsh and Ritter, 1981), CD73 and CD105 (Alt et al., 2011) with MSCs (Halfon et al., 2011). These fibroblasts are frequently co-isolated when establishing primary cell cultures and can overgrow a cell culture rapidly (linge et al., 1989). Therefore, a confirmation of a genuine MSC culture and not fibroblasts is a prerequisite. Instead of using phenotypic analysis, gene expression profiling of cells could be a better approach to characterise the cells under investigation and confirm their MSC identity. Phenotypical evaluation of MSCs is mostly performed by FACS analysis and could therefore be considered as the gold standard. Nevertheless, FACS results only identify the number of cells in a sample that express a phenotypic marker in a fairly binary way. Additionally, gene expression can be reliably measured by quantitative PCR (qPCR). Cells with the same phenotypic profile could therefore be distinguished by their transcriptomic profile. In a previous study (Zhang et al., 2019), we studied bone marrow derived MSCs (BM-MSCs) cultured on surfaces with varying topography (flat versus fibrous) and chemistry (aminated versus pristine). RNA-Seq data from these cultures were used to generate their transcriptomic profiles and identify the effect of topography and chemistry on the expression of 177 previously reported MSC markers (several are reviewed in (Lv et al., 2014; Uder et al., 2018)). The gene expressions of these markers were processed through network analysis to determine the optimal cluster distribution, being organized into 4 clusters to achieve the optimal network integrity (Zhang et al., 2019). From these clusters we selected several genes to identify MSC specific gene expression biomarkers. In another publication (Brinkhof et al., 2018), we identified several reference genes suitable for gene expression normalisation after umbilical cord derived MSCs (UC-MSCs) and BM-MSCs were cultured in 2D on tissue culture plate (TCP) or 3D on scaffolds. A selection of these previously isolated RNA samples (Brinkhof et al., 2018) has been used to further identify genes stably expressed in MSCs depending on topography. In addition to these samples, RNA has been extracted from two commercially obtained primary BM-MSCs, hTERT immortalised MSCs (MSC-hTERT) and fibroblasts, cultured at different oxygen levels. Together with the previously extracted samples (Brinkhof et al., 2018), these newly isolated samples have been screened for the expression levels of selected marker genes to reliably characterise hMSCs and enable differentiation from fibroblasts. Amongst these tested genes, *ALCAM* was identified as upregulated in all MSC sample groups compared to fibroblasts. The expression levels positively correlated to those of *ENG* (CD105), though *ALCAM* was more specific for MSCs.

## 2 Materials and methods

### 2.1 Cell culture

Two sources of primary and one tert-immortalised cell line of bone marrow derived hMSCs were acquired from Lonza (referred to as MSC-L, PT-2501, Slough, UK), PromoCell (Referred to as MSC-P, C-12974, Heidelberg, Germany), and a collaborating laboratory prepared using the method described in (Mihara et al., 2003) (referred to as MSC-T), respectively. The establishment of human umbilical cord derived MSCs (referred to as MSC-U) has been described in detail before (Brinkhof et al., 2018). Human dermal fibroblast (hDF) was acquired from ThermoFisher (C0135C, Hemel Hempstead, UK). Cells were cultured in incubators maintained at 5% CO_2_ in air. Cells under hypoxia exposure (O_2_-levels are indicated in Supplemental Table 1) were cultured in a Hypoxystation-H35 (Don Whitley Scientific, Bingley, UK) supplemented with 5% CO_2_. Cells were transferred into the Hypoxystation immediately after seeding. Cells were cultured in a serum-free, xeno-free media, MSCs NutriStem (Biological Industries, Cromwell, USA) which was changed every 3 days. Cells were cultured in 6-well cell culture plates (Costar, ThermoFisher) with a seeding density of 5000 cells/cm^2^. The passage number of 4–6 was used for the two sources of primary hMSCs, passage number of 6–10 was used for the immortalised hMSCs and passage number of 10–12 was used for hDF. Further sample details can be found in Supplemental Table 1.

### 2.2 RNA extraction and cDNA conversion

RNA extraction from the MSC-U samples and some of the MSC-T samples cultured in 2D (tissue culture plate) and 3D (fibrinogen scaffolds or polycaprolactone-poly[N-isopropylacrylamide] beads) used in this study has been described previously (Brinkhof et al., 2018) (Supplemental Table 1). The hMSCs and hDFs cultured under different oxygen levels (Supplemental Table 1) were harvested with trypsin solution (59418C, Sigma-Aldrich, Dorset, UK) and collected into a pellet. Trizol (11596-018, ThermoFisher) was added onto the cell pellet and resuspended. Samples that were not processed immediately were stored under −80 °C for future extraction. For RNA extraction, 1-bromo-3-cholopropane (B9673, Sigma-Aldrich) was added into the mix, incubated, centrifuged, and the upper layer, containing the RNA, transferred to a new tube. To purify the RNA, a mixture of phenol-chloroform-isoamyl alcohol (77619, Sigma-Aldrich) was added into the solution, incubated, centrifuged, and the upper phase was transferred to a new tube. To precipitate the RNA, 2-propanol (I9516, Sigma-Aldrich) was added, incubated, centrifuged and the supernatant was discarded. RNA pellets were washed in 75% ethanol (ThermoFisher) and air-dried before resuspending in H_2_O (ThermoFisher). RNA concentration was measured using the Nanodrop One (ThermoFisher) and quality was assessed using an Agilent 2100 Bioanalyzer (Agilent Technologies, Santa Clara, CA, USA). A detailed procedure for the RNA extraction can be found in (Brinkhof et al., 2006). RNA was stored at −80 °C or used immediately for reverse transcriptase reactions. To generate cDNA, 1 μg of RNA was used for initial elimination of genomic DNA (QuantiTect Reverse Transcription Kit, Qiagen, Manchester, UK) in 14 μl reaction volume. Genomic DNA elimination reaction was performed at 42 °C for 5 min. Subsequently, a mixture containing Reverse Transcriptase (RT), a mix of oligo-dT and random primers, and RT buffer was added to a final volume of 20 μl. Reverse-transcription reaction was performed at 42 °C for 30 min followed by an inactivation step at 95 °C for 5 min. All procedures were performed per manufacturers protocol (Qiagen) in a Rotor-Gene 6000 (Corbett Research, Mortlake, Australia). All cDNA samples were stored at −20 °C until further use.

### 2.3 Quantitative PCR and data analysis

All cDNA samples were measured in duplicate in a 96 well plate covered with adhesive seals. To fit all samples, two plates (A and B) were used per gene and both plates contained standards. These standards were generated by diluting an MSC sample 5-fold until S7. Plate A contained 40 samples and plate B the remaining samples. For all measurements 1 μl cDNA template per 20 μl final reaction volume was used on an Applied Biosystems StepOnePlus Real-time PCR system (Applied Biosystems, Warrington, UK) based on the SyGreen intercalating dye and a passive reference ROX (PCR Biosystems, London, UK). All primers had a final concentration of 400 nM each. Reactions started with 3 min at 95 °C, followed by 40 cycles of 15 s at 95 °C and 30 s at Tm. This reaction was followed by a melting curve, stepwise increasing temperature each 15 s by 0.5 °C, ranging from 65 °C to 95 °C. Recommended Tm was used for previously published primers or optimal gene specific Tm was determined using a temperature gradient for newly developed primer sets using the same standards as for the actual measurements (Table 1). LinRegPCR (Ramakers et al., 2003) version 2016.1 was used for baseline correction (Ruijter et al., 2009) and quantification cycle (Cq) values were loaded into qBase Plus (Hellemans et al., 2007) version 3.2 for relative quantity and correlation (Pearson and Spearman) analysis. After amplification efficiency determination the five primer sets selected for gene expression normalisation (*PPIA*, *PUM1*, *TBP*, *TFRC*, and *YWHAZ*) were analysed for their suitability as reference gene using geNorm (Vandesompele et al., 2002).

**Table 1:** Primer details for selected genes.

Gene symbol^a^ (gene ID)	NCBI Ref Seq^b^	Full gene name^a^	Primer sequence 5′→3′^c^	Amplicon length (bp)	Tm (°C)
ALCAM	NM_001627	Activated leukocyte cell adhesion molecule	F: CATACCTTGCCGACTTGACG	91	63
(214)			R: GAAGGCAATAAATACTGGGGAGC		
BSG	NM_001728	Basigin (Ok blood group)	F: GAACACATCAACGAGGGGGA	154	64
(682)			R: CCTGCGAGGAACTCACGAAG		
CD59	NM_203330	CD59 molecule (CD59 blood group)	F: TGCGTGTCTCATTACCAAAGC	207	64
(966)			R: GGAGTCACCAGCAGAAGAACT		
CD63	NM_001780	CD63 molecule	F: TTCAACGAGAAGGCGATCCA	179	63
(967)			R: CCCTACATCACCTCGTAGCC		
CLIC1	NM_001287593	Chloride intracellular channel 1	F: AGTTTTTGGATGGCAACGAGC	177	64
(1192)			R: CTGGACAGGTGGAAGCGAAT		
CLIC4	NM_013943	Chloride intracellular channel 4	F: GTGTGACGACTGTTGACCTGA	211	63
(25932)			R: GCAAAGATGTCCATTCCAGCAG		
EDIL3	NM_005711	EGF like repeats and discoidin domains 3	F: TACCCAAGGAGCCAAGAGGA	250	62
(10085)			R: GCCAAGAAGTTCCATTCGCA		
ENG	NM_001114753Brinkhof et al., 2018	Endoglin	F: CCCAAAACCGGCACCCTCA	238	64
(2022)			R: TGGGGGAACGCGTGTGC		
EPHA2	NM_004431	EPH receptor A2	F: CTGCCAGTGTCAGCATCAAC	141	60
(1969)			R: TCTTGCGGTAAGTGACCTCG		
FN1	NM_212482	Fibronectin 1	F: ATTCCAATGGTGCCTTGTGC	214	59
(2335)			R: TCCCACTGATCTCCAATGCG		
IGFBP7	NM_001553	Insulin like growth factor binding protein 7	F: GTCCTTCCATAGTGACGCCCC	232	66
(3490)			R: GATACCAGCACCCAGCCAGT		
ITGA1	NM_181501	Integrin subunit alpha 1	F: ATGGGTGCTTATTGGTTCTCCG	199	64
(3672)			R: TCCTCCATTTGGGTTGGTGAC		
LAMP1	NM_005561	Lysosomal associated membrane protein 1	F: GGTGAAAAATGGCAACGGGAC	112	59
(3916)			R: TGATGGCAGGTCAAAGGTCA		
LRRC59	NM_018509	Leucine rich repeat containing 59	F: GCTCAGGCGTCGTCGTTT	240	63
(55379)			R: CAGGATGGTGGCCTTTGGAA		
MCAM	NM_006500	Melanoma cell adhesion molecule	F: GTCCACATTCAGTCGTCCCA	238	60
(4162)			R: GGTCCCCTTCCTTCAGCATT		
NECTIN2	NM_002856	Nectin cell adhesion molecule 2	F: GCCAAAGAGACTCAGGTGTCA	217	64
(5819)			R: GGCCGAGGTACCAGTTGTC		
NT5E	NM_002526Brinkhof et al., 2018	5′-nucleotidase ecto	F: GGCTGCTGTATTGCCCTTTG	175	64
(4907)			R: TACTCTGTCTCCAGGTTTTCGG		
PPIA ^d^	NM_021130Su et al., 2016	Peptidylprolyl isomerase A	F: GTCAACCCCACCGTGTTCTT	97	60
(5478)			R: CTGCTGTCTTTGGGACCTTGT		
PUM1 ^d^	NM_001020658Brinkhof et al., 2018	Pumilio RNA binding family member 1	F: CAGGACATTCACAGACACCA	196	66
(9698)			R: CGCAAACGAGAGGAAGAGA		
TBP^d^	NM_003194Brinkhof et al., 2018	TATA-box binding protein	F: ATCAGAACAACAGCCTGCC	113	64
(6908)			R: GGTCAGTCCAGTGCCATAAG		
TFRC^e^	NM_003234Brinkhof et al., 2018	Transferrin receptor	F: CTGGCTCGGCAAGTAGATG	234	62
(7037)			R: TGCCAGTCTCTCACACTCA		
THY1	NM_006288Brinkhof et al., 2018	Thy-1 cell surface antigen	F: AGCATCGCTCTCCTGCTAAC	230	65
(7070)			R: CTGGTGAAGTTGGTTCGGGA		
TLN1	NM_006289	Talin 1	F: ATTATGCAGGTATTGCAGCTCG	242	64
(7094)			R: AGCCTGGGTCACTGCTTTAG		
TMEM47	NM_031442	Transmembrane protein 47	F: TCATTGCATTCCTGGTGGGT	244	64
(83604)			R: GGGTTCAGGCAATAAAGGATGG		
YWHAZ^d^	NM_145690Brinkhof et al., 2018	Tyrosine 3-monooxygenase/tryptophan 5-monooxygenase activation protein zeta	F: TCATCTTGGAGGGTCGTCT	180	64
(7534)			R: GACTTTGCTCTCTGCTTGTG		

a Provided by HUGO Gene Nomenclature Committee (HGNC).

b Reference of original first publication given if applicable.

c F = Forward primer; R = Reverse primer.

d Candidate reference gene.

e Used as candidate reference gene and as gene of interest.

### 2.4 Statistical analysis

All statistical calculations are described in detail in the referred manuscripts (Hellemans et al., 2007; Ramakers et al., 2003; Ruijter et al., 2009; Vandesompele et al., 2002). Relative quantities per sample as calculated in qBase plus were exported to Excel for further statistical analysis. The geometric mean and the standard error of the mean (SEM) were calculated per cell type (hDF, MSC-L, MSC-P, MSC-T, and MSC-U). A student *t*-test was used to identify statistically different gene expression levels (*p* < 0.05) between the cell types. For the relative expression figures in the manuscript all data have been normalised to fibroblasts. The original relative expression data with their SEM indicated are shown in Supplemental Fig. 1.

## 3 Results and discussion

### 3.1 Gene expression qualification

MSCs cultured under various conditions such as topography (2D vs. 3D) (Brinkhof et al., 2018) and oxygen levels were analysed for their gene expression levels. When establishing and culturing MSCs, fibroblasts are the most frequent contaminating cell type. Therefore, it is important to distinguish genuine MSCs from fibroblasts. After baseline determination (Ruijter et al., 2009), Cq-values were exported to excel for further analysis using qBase plus (Hellemans et al., 2007). Amplification efficiencies were determined for 25 genes using Cq-values from the standards run on each plate. All efficiencies were between 92.7%–107.0% (1.927–2.070) and regressions r^2^ ≥ 0.99 (Table 2), indicating good sample quality and qPCR reaction.

**Table 2:** Gene specific qPCR run details.

Gene	Slope		Y-intercept		Efficiency		r^2^
	Value	sd	Value	sd	Value	sd	
ALCAM	−3.466	0.062	25.123	0.101	1.943	0.023	1.00
BSG	−3.391	0.084	23.929	0.150	1.972	0.033	0.99
CD59	−3.498	0.050	22.511	0.104	1.931	0.018	1.00
CD63	−3.501	0.027	18.691	0.060	1.930	0.010	1.00
CLIC1	−3.452	0.053	21.699	0.116	1.948	0.020	1.00
CLIC4	−3.395	0.049	23.490	0.076	1.970	0.019	1.00
EDIL3	−3.378	0.058	25.314	0.122	1.977	0.023	0.99
ENG	−3.248	0.068	26.109	0.127	2.032	0.030	0.99
EPHA2	−3.379	0.102	30.045	0.123	1.977	0.041	0.99
FN1	−3.511	0.036	19.996	0.072	1.927	0.013	1.00
IGFBP7	−3.477	0.080	23.239	0.153	1.939	0.030	0.99
ITGA1	−3.247	0.067	27.299	0.077	2.032	0.030	1.00
LAMP1	−3.230	0.088	23.434	0.168	2.040	0.040	0.99
LRRC59	−3.438	0.099	25.379	0.111	1.954	0.038	0.99
MCAM	−3.490	0.088	26.839	0.147	1.934	0.032	0.99
NECTIN2	−3.229	0.071	25.966	0.120	2.040	0.032	0.99
NT5E	−3.403	0.061	23.873	0.126	1.967	0.024	0.99
PPIA	−3.454	0.068	20.925	0.157	1.948	0.025	0.99
PUM1	−3.165	0.044	26.698	0.069	2.070	0.021	1.00
TBP	−3.216	0.115	27.849	0.111	2.046	0.052	0.99
TFRC	−3.316	0.033	24.214	0.053	2.002	0.014	1.00
THY1	−3.435	0.106	27.653	0.114	1.955	0.041	0.99
TLN1	−3.441	0.059	24.990	0.092	1.953	0.022	1.00
TMEM47	−3.453	0.063	26.875	0.094	1.948	0.024	1.00
YWHAZ	−3.372	0.057	22.589	0.114	1.980	0.023	1.00

**Fig. 1:**
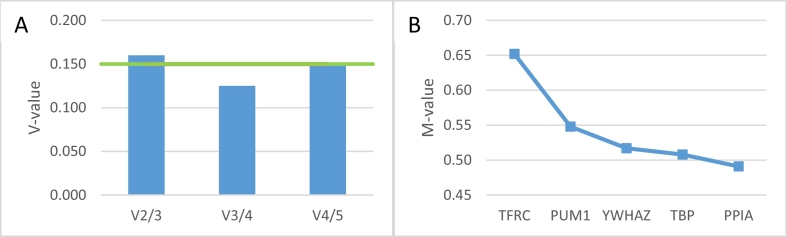
GeNorm analysis. A) Candidate reference gene (RG) variability (V-values) indicating the variability between 2 or 3 RGs (V2/3), 3 or 4 RGs (V3/4), and 5 or 6 RGs (V4/5). The green line indicates the cut-off value (0.150). B) Average expression stability of remaining candidate reference genes (M-value). (For interpretation of the references to colour in this figure legend, the reader is referred to the web version of this article.)

A selection of five genes (*YWHAZ*, *TFRC*, *TBP*, *PUM1*, and *PPIA*) has been made from a panel of 12 previously-validated candidate reference genes (Brinkhof et al., 2018) to analyse for their suitability as gene expression normalisers in this sample set. GeNorm analysis indicated the optimal number of reference genes was three (V-value, Fig. 1A) and the advised gene targets for normalisation were *TBP*, *YWHAZ*, and *PPIA* with a medium reference gene stability (0.5 < M-value <1.0) (Fig. 1B). Since the sample set consisted of several MSC cell lines and fibroblasts this stability was expected from such a heterogeneous sample set (Hellemans et al., 2007). Further analysis was performed selecting the advised genes (*TBP*, *YWHAZ*, and *PPIA*) as normalisers.

### 3.2 ISCT MSC gene expression analysis

**Fig. 2:**
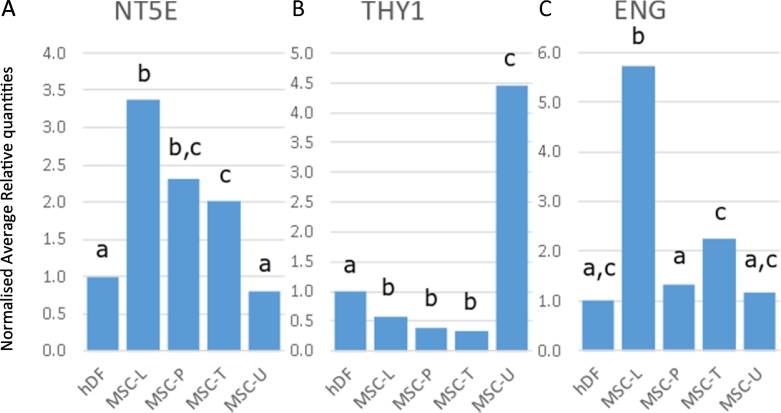
Normalised relative expression in MSCs for ISCT markers. Relative quantities (geometric means) for fibroblasts (hDF, set at 1) and MSCs (MSC-L: MSCs supplied by Lonza, MSC-P: MSCs supplied by PromoCell, MSC-T: hTERT immortalised MSCs, MSC-U: Umbilical cord derived MSCs) are given for A) *NT5E* (CD73), B) *THY1* (CD90), and C) *ENG* (CD105). For each graph, different letters indicate significant (*p* < 0.05) differences.

Statistical analysis for the common MSC markers 5′-Nucleotidase Ecto *NT5E* (CD73), Cell Surface Antigen *THY1* (CD90), and Endoglin *ENG* (CD105), indicated that the expression for *NT5E* was significantly higher in BM-MSCs (MSC-L, MSC-P, and MSC-T) compared to fibroblasts (hDF), whereas the MSC-U showed no significant expression level difference (Fig. 2A). For *THY1*, the MSC-U expression level was almost 4.5-fold higher than in fibroblasts, whereas the expression in BM-MSC was lower by >2-fold (Fig. 2B). *ENG* gene expression levels were similar for MSC-P and MSC-U, whereas the expression levels in MSC-L and MSC-T were increased by 5.7-fold and 2.2-fold, respectively (Fig. 2C), compared to fibroblasts. These results together, indicate these ISCT markers (Dominici et al., 2006) are not very suitable as general MSC specific markers when using gene expression analysis, in particular when comparing with fibroblasts (Alt et al., 2011; Halfon et al., 2011). Another 18 genes, including *TFRC* as it has also been suggested as a MSC selection marker (Jeong et al., 2007; Zuk et al., 2002), have been analysed for their expression levels in the MSCs and fibroblasts (Supplemental Fig. 1).

### 3.3 *ALCAM* expression and its feasibility as a biomarker in MSCs

**Fig. 3:**
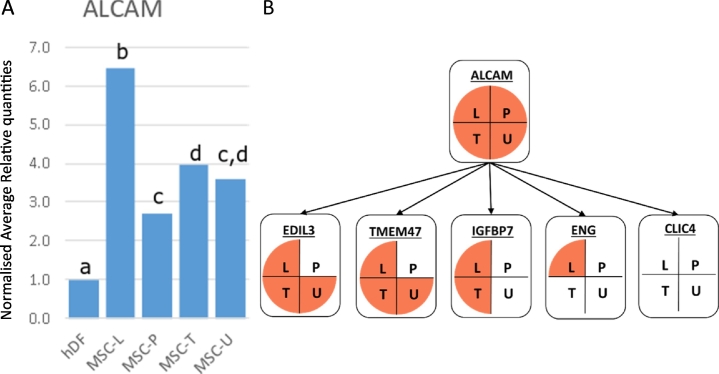
*ALCAM* expression. A) Normalised relative quantities for fibroblasts (hDF, set at 1) and MSCs (MSC-L: MSCs supplied by Lonza, MSC-P: MSCs supplied by PromoCell, MSC-T: hTERT immortalised MSCs, MSC-U: Umbilical cord derived MSCs) are given for *ALCAM*. Different letters indicate significant (*p* < 0.05) differences. B) Significant differential expression for MSCs (L: MSCs supplied by Lonza, P: MSCs supplied by PromoCell, T: hTERT immortalised MSCs, U: Umbilical cord derived MSCs) with fibroblasts (orange) for *ALCAM* and those gene expression levels significantly correlated (*p* < 0.05, *r* > 0.5) to *ALCAM*.

Activated leukocyte cell adhesion molecule, *ALCAM*, a type-I transmembrane protein, belonging to the immunoglobulin superfamily (Bowen et al., 1995) a.k.a. CD166, was the only tested gene to be expressed at higher levels in both BM-MSCs and UC-MSCs than in fibroblasts (Fig. 3A). The ALCAM protein has been identified as a possible human MSC surface marker (Bruder et al., 1998; Mareschi et al., 2009; Mildmay-White and Khan, 2017), although its role in MSCs seems to be undetermined (Moraes et al., 2016). Cells expressing CD166 on their membrane are reported to have favourable chondrogenic differentiation capacity (Jonitz et al., 2011). ALCAM has also been implicated in various pathologies such as multiple sclerosis (Wagner et al., 2014), heart disease (Iolyeva et al., 2013) and cancer (von Lersner et al., 2019; Yavuz et al., 2018). The molecule is also present in hematopoietic stem cells (Jeannet et al., 2013), cancer stem cells (Manhas et al., 2016) and intestinal stem cells (Wang et al., 2013). On an mRNA level, *ALCAM* has only been identified as a human MSC marker in a few papers. In these papers, human BM-MSCs were described as being positive for *ALCAM* gene expression (Rallapalli et al., 2009). Its expression was analysed in MSC-like progenitors (MPC) derived from mild and severe osteoarthritic tibial plateaus without significant differential expression (Mazor et al., 2017). In a report comparing *ALCAM* expression in UC and dental pulp (DP) derived MSCs, qPCR indicated an 8-fold higher expression of the gene in UC-MSCs (Kang et al., 2016). A comparison of BM-MSCs and adipose derived MSCs (AD-MSCs) showed no significant differential *ALCAM* expression (Winkler et al., 2016). Microarray gene expression analysis revealed similar *ALCAM* levels in BM-MSC and osteosarcoma (OS) derived MSCs (Brune et al., 2011) and placenta (PL) derived MSCs (Brooke et al., 2008). When single MSCs from unexpanded bone biopsies from healthy donors and multiple myeloma (MM) patients were compared, *ALCAM* was expressed at similar levels, though in only 80% of the cells (Mehdi et al., 2019). Our data, together with these previous published results, indicate the stable and consistent *ALCAM* expression amongst MSCs regardless the tissue they are originally derived from. In other species *ALCAM* is also used as a gene expression marker for MSCs (Calloni et al., 2014; Kovac et al., 2017). In horses, *ALCAM* expression levels were similar to *ENG* and were not differentially expressed between BM and AD derived MSCs (Ranera et al., 2011). Cultures of porcine amniotic membrane derived MSCs showed a reduction in *ALCAM* expression after passage 3 (Lange-Consiglio et al., 2015). Rabbit (rb) amniotic fluid (AF) derived MSCs (Kovac et al., 2017) were also positive for *ALCAM* gene expression whereas rbBM-MSCs were negative (Jin et al., 2014). The heterogeneous sample set used in this study represents different time points, culture dimension and oxygen levels during culture (Supplemental Table 1), the stable and high expression of *ALCAM* indicates this gene could serve as a robust marker for MSCs in gene expression analysis. Further analysis indicated a strong correlation with several other genes (Fig. 3B, Supplemental Table 2). Amongst these was *ENG*, suggesting *ALCAM* could replace *ENG* in a panel of genes for the accurate identification and characterisation of MSCs in general. In particular, since *ALCAM* is expressed at higher levels in all tested MSCs than in the fibroblasts (Fig. 3), which is not the case for *ENG* (Fig. 2C). In previous work, it has already been suggested that other genes would be more specific in representing the mesenchymal signature than THY1 (Roson-Burgo et al., 2016). Future studies need to be designed to provide further evidence on whether *ALCAM* is a more preferred choice in replacing *THY1* and *ENG*.

### 3.4 *CLIC1* as a BM-MSC biomarker

**Fig. 4:**
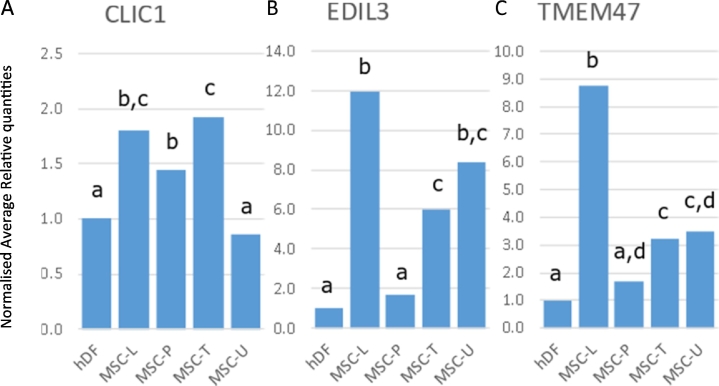
Normalised relative expression in MSCs for transcriptomics markers *CLIC1*, *EDIL3*, and *TMEM47*. Normalised relative quantities for fibroblasts (hDF, set at 1) and MSCs (MSC-L: MSCs supplied by Lonza, MSC-P: MSCs supplied by PromoCell, MSC-T: hTERT immortalised MSCs, MSC-U: Umbilical cord derived MSCs) are given for A) *CLIC1*, B) *EDIL3*, and C) *TMEM47*. For each graph, different letters indicate significant (*p* < 0.05) differences.

The chloride intracellular channel 1; CLIC1, was originally identified as NCC27 (Valenzuela et al., 1997). Its expression varies depending on cell type, distributed from intracellular vesicular to intranuclear (Ashley, 2003; Liao and Chang, 2012). MSCs differentiated into osteoblasts showed increased *CLIC1* expression, whereas adipogenic differentiation abolished *CLIC1* expression (Yang et al., 2009). In our study, *CLIC1* was expressed at higher levels in BM-MSCs than in MSC-U or fibroblasts (Fig. 4A). Relative expression levels for *CLIC1* were very similar to those for *NT5E* (Fig. 2A) which confirms our previous finding that these genes belong to the same genetic cluster (Zhang et al., 2019). Expression differences between fibroblasts and BM-MSC samples were greater for *NT5E* than for *CLIC1* indicating a preferential use of *NT5E* for BM-MSC identification. Its paralog, *CLIC4*, correlated with *ALCAM* (Fig. 3B, Supplemental Table 2), though was not significantly different from fibroblasts for all MSC groups (Supplemental Fig. 1).

### 3.5 Gene expression of *EDIL3* and *TMEM47* could aid in hMSC identification

Two other genes correlated to *ALCAM* were *EDIL3* and *TMEM47* (Fig. 3B). The integrin ligand EGF Like Repeats And Discoidin Domains 3, encoded by *EDIL3* (a.k.a. DEL1), is a protein playing an important role in mediating angiogenesis and is regulated upon hypoxia or vascular injury (Ho et al., 2004; Penta et al., 1999). It promotes adhesion of endothelial cells through interaction with the alpha-v/beta-3 integrin receptor (Hidai et al., 1998; Penta et al., 1999). Dermal MSCs from psoriatic skin lesions contained higher levels of EDIL3 mRNA as well as protein compared to their healthy counterparts (Niu et al., 2016). The Transmembrane Protein 47 gene, *TMEM47*, encodes a member of the PMP22/EMP/claudin protein family. The protein, localized to the ER and plasma membrane, regulates cell junction organization in epithelial cells (Christophe-Hobertus et al., 2001). Using microarray studies, the gene has been detected in MSCs and fibroblasts (Jaager et al., 2012; Roson-Burgo et al., 2016). Although expressed at higher levels in all MSC samples than in fibroblasts, both *EDIL3* and *TMEM47* were not significantly different from fibroblasts in the MSC-P samples (Fig. 4B and C). Expression of *EDIL3* in MSC-P was high at the beginning (day 0, day 1) and end (day 7) of culture and reduced to even below the fibroblast levels after 3 and 5 days in culture.

### 3.6 ITGA1 as a potential negative biomarker for BM-MSCs

**Fig. 5:**
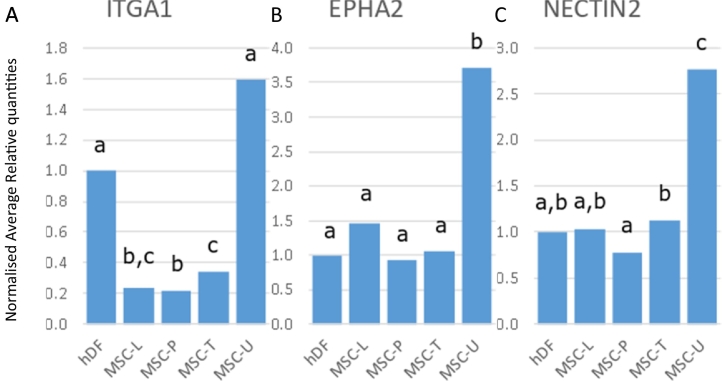
Normalised relative expression in MSCs for *ITGA1*, *EPHA2*, and *NECTIN2*. Relative quantities for fibroblasts (hDF, set at 1) and MSCs (MSC-L: MSCs supplied by Lonza, MSC-P: MSCs supplied by PromoCell, MSC-T: hTERT immortalised MSCs, MSC-U: Umbilical cord derived MSCs) are given for A) *ITGA1*, B) *EPHA2*, and C) *NECTIN2*. For each graph, different letters indicate significant (*p* < 0.05) differences.

Integrin alpha 1 (ITGA1), a.k.a. the very late activation protein VLA1 or CD49a, associates with the beta-1 chain (ITGB1) to form a heterodimer that functions as a dual laminin/collagen receptor in neural cells and hematopoietic cells (Briesewitz et al., 1993). Surface property has been suggested to play a role in osteogenic differentiation of MSCs increasing *ITGA1* expression (Olivares-Navarrete et al., 2011). ITGA1 has been used to isolate BM-MSCs efficiently in previous studies (Deschaseaux and Charbord, 2000; Rider et al., 2007; Stewart et al., 2003), though fibroblasts do express ITGA1 as well (Gardner, 2014). In our study, BM-MSCs showed significant lower expression levels of *ITGA1* than fibroblasts or UC-MSCs (Fig. 5A) and could possibly be used as a negative marker for BM-MSC gene expression studies. It could also aid in the distinction between BM-MSCs and fibroblasts. Apart from being not significant for MSC-U, the relative expression profile of *ITGA1* for all sample types was very similar to that of *THY1* (Comparing Fig. 5A with Fig. 2B).

### 3.7 Positive biomarkers for UC-MSCs

The UC-MSC samples expressed Ephrin type-A receptor 2 (*EPHA2)* and the Nectin cell adhesion molecule *NECTIN2* at higher levels than any of the other cell lines (Fig. 5B and C). EPHA2 is a member of the EPH receptor tyrosine kinases. It binds to membrane-bound ephrin-A family ligands on adjacent cells resulting in contact-dependent bidirectional signalling into neighbouring cells. EPHA2 has only recently been reported as a candidate biomarker for PL-MSCs, UC-MSCs (Shen et al., 2015), AD-MSCs and BM-MSCs (Holley et al., 2015). NECTIN2 is a single-pass type I membrane glycoprotein with two Ig-like C2-type domains and an Ig-like V-type domain. Also known as HveB or PRR2, the protein is mainly associated with virus entry into cells and is expressed on certain fibroblasts (Eberlé et al., 1995; Lopez et al., 2000; Warner et al., 1998). NECTIN2 has been reported to be expressed on MSCs (Spaggiari et al., 2006) involved in the activation of Natural Killer (NK) cells (Poggi and Giuliani, 2016) and subsequent lysis of the MSCs (Crop et al., 2011). A similar expression level profile was found for *EPHA2* and *NECTIN2*, although, in contrast to *ITGA1* and *THY1*, BM-MSCs were not significantly different from fibroblasts. Even *TFRC*, a candidate reference gene in MSCs (Brinkhof et al., 2018; Su et al., 2016) as well as a selection marker for MSCs (Jeong et al., 2007; Zuk et al., 2002), showed similar expression levels for the MSCs and fibroblasts as *EPHA2* and *NECTIN2* (Supplemental Fig. 1) though no consistent significant expression differences could be identified.

## 4 Conclusion

Mesenchymal stromal cells are a valuable cell type for regenerative medicine (RM) and tissue engineering (TE). MSCs can be easily extracted from several tissues in the body e.g. extra-embryonic tissue, fat or bone marrow. Unfortunately, often, the majority of the extracted cells are fibroblasts and the MSCs are only a small portion. To obtain sufficient cells for RM or TE, these MSCs need to be expanded in vitro. As fibroblast can overgrow a cell culture rapidly, it is of utmost importance that these fibroblasts are eliminated from culture. The ISCT has proposed three minimal phenotypical markers to identify MSCs (Dominici et al., 2006). These markers are also expressed on fibroblasts. A transcriptomics approach could identify differences in expression for these ISCT markers or other genes previously identified as surface markers for MSCs. However, whole transcriptome analysis has not been feasible for routine practice in tissue culture laboratories. Instead, qPCR has continuously been one of the commonly used techniques in MSCs studies. Here, we identified *ALCAM* as a candidate gene for the identification of genuine MSCs in contrast to fibroblasts. We also confirmed our previous finding of a genetic positive correlation between *ALCAM* and *ENG* (CD105) expressions (Zhang et al., 2019), indicating only one of these genes needs to be tested to confirm MSC identity. The superior specificity, sensitivity and reliability, favours the use of *ALCAM* over *ENG*. Additional genes that could be used are *EDIL3* and *TMEM47*. Both *CLIC1* and *NT5E* were more specific for BM-MSCs than UC-MSCs and *EPHA2* or *NECTIN2* were more specific for UC-MSCs. These genes, in particular *ALCAM*, could aid in the characterisation of MSCs and distinguish them from fibroblasts in cell culture and, therefore, improve their application in tissue engineering and regenerative medicine.

## Declaration of competing interest

The authors declare that they have no known competing financial interests or personal relationships that could have appeared to influence the work reported in this paper.

## Appendix A Supplementary data

Supplementary material Image 1
