# Supplementary material for: ALCAM (CD166) as a gene expression marker for human mesenchymal stromal cell characterisation
Source: Gene X. 2020 Mar 14;5:100031. doi: 10.1016/j.gene.2020.100031 (PMC7285916; doi:10.1016/j.gene.2020.100031)
Supplement: Supplementary file 1 — Supplementary material [file mmc1.docx]

*Supplemental Files:*


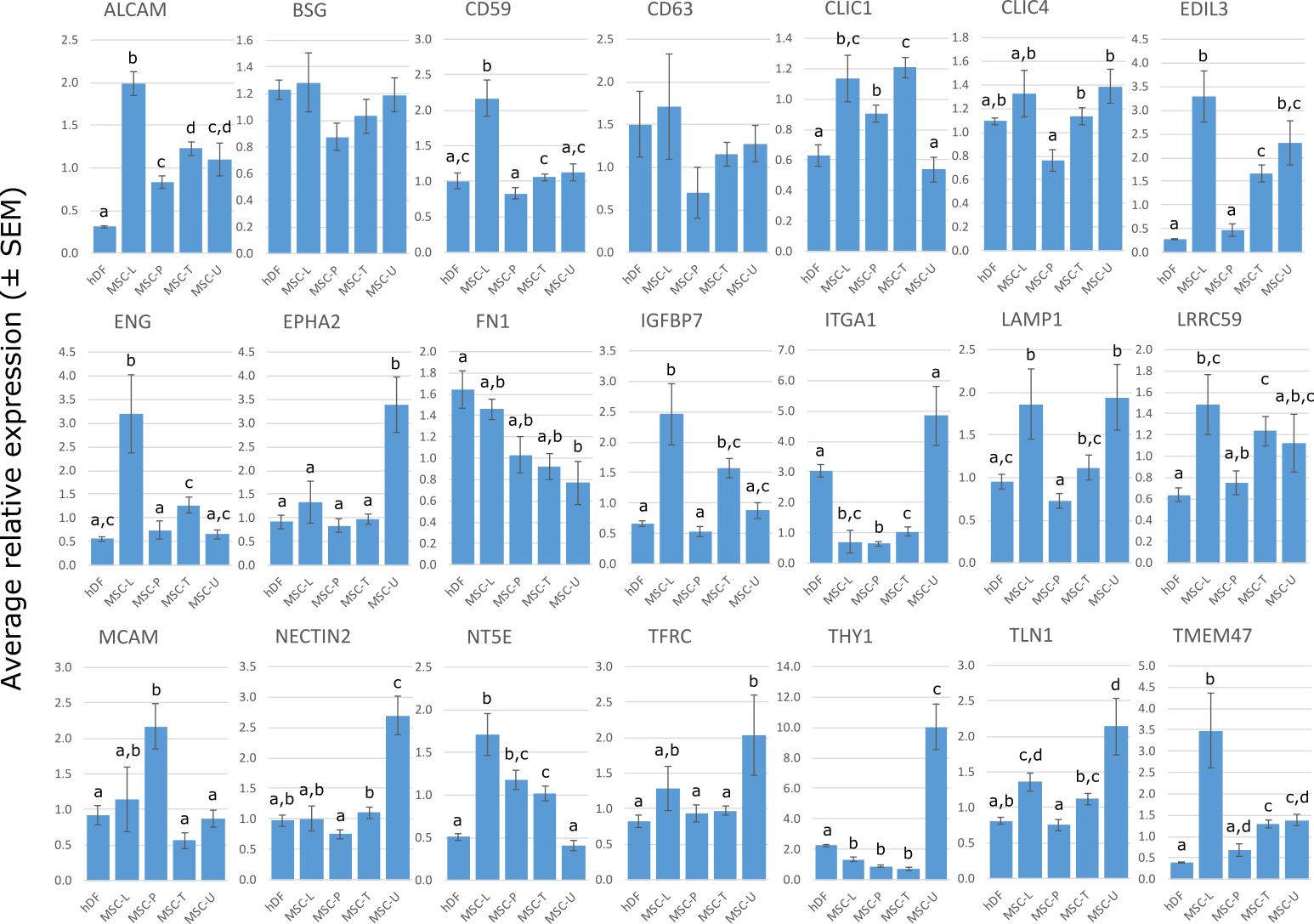


**Supplemental Figure 1. Relative quantities for all GOI.** 21 genes have been analysed for their gene expression in fibroblasts (hDF; n=4), MSCs obtained from Lonza (MSC-L; n=3), MSCs obtained from PromoCell (MSC-P; n=25), TERT immortalised MSCs (MSC-T; n=34), and primary umbilical cord derived MSCs (MSC-U; n=4). If significantly different (p<0.05) after two-tailed t-test this is indicated with different letters in the gene specific graph. Whiskers represent standard error of the mean (SEM).

**Supplemental Table 1. RNA samples.**

| Sample ^a^ | Culture days | Culture dimension | O2 % | Cell type |
| --- | --- | --- | --- | --- |
| F1 | 5 | 2D | 2 | hDF |
| F2 | 5 | 2D | 20 | hDF |
| F3 | 5 | 2D | 20 | hDF |
| F4 | 5 | 2D | 20 | hDF |
| L1 | 1 | 2D | 1 | MSC-L |
| L2 | 3 | 2D | 20 | MSC-L |
| L3 | 3 | 2D | 20 | MSC-L |
| P1 | 5 | 2D | 0.1 | MSC-P |
| P2 | 3 | 2D | 1 | MSC-P |
| P3 | 3 | 2D | 1 | MSC-P |
| P4 | 5 | 2D | 2 | MSC-P |
| P5 | 5 | 2D | 2 | MSC-P |
| P6 | 5 | 2D | 2 | MSC-P |
| P7 | 3 | 2D | 20 | MSC-P |
| P8 | 3 | 2D | 20 | MSC-P |
| P9 | 5 | 2D | 20 | MSC-P |
| P10 | 5 | 2D | 20 | MSC-P |
| P11 | 5 | 2D | 20 | MSC-P |
| P12 | 5 | 2D | 20 | MSC-P |
| P13 | 5 | 2D | 20 | MSC-P |
| P14 | 5 | 2D | 20 | MSC-P |
| P15 | 7 | 2D | 20 | MSC-P |
| P16 | 7 | 2D | 20 | MSC-P |
| P17 | 7 | 2D | 20 | MSC-P |
| P18 | 1 | 2D | 5 | MSC-P |
| P19 | 1 | 2D | 5 | MSC-P |
| P20 | 1 | 2D | 5 | MSC-P |
| P21 | 3 | 2D | 5 | MSC-P |
| P22 | 3 | 2D | 5 | MSC-P |
| P23 | 0 | 2D | 20 | MSC-P |
| P24 | 0 | 2D | 20 | MSC-P |
| P25 | 0 | 2D | 20 | MSC-P |
| 3D Fib-Alg 2^a^ | 3 | 3D | 20 | MSC-T |
| Cells 12W 3^a^ | 3 | 2D | 20 | MSC-T |
| Cells 48W 1^a^ | 3 | 2D | 20 | MSC-T |
| Cells 48W 3^a^ | 3 | 2D | 20 | MSC-T |
| BEADS 1^a^ | 3 | 3D | 20 | MSC-T |
| BEADS 2^a^ | 3 | 3D | 20 | MSC-T |
| BEADS 3^a^ | 3 | 3D | 20 | MSC-T |
| 3D Fib 1^a^ | 3 | 3D | 20 | MSC-T |
| 3D Fib 2^a^ | 3 | 3D | 20 | MSC-T |
| 3D Fib 3^a^ | 3 | 3D | 20 | MSC-T |
| T11 | 0.1 | 2D | 1 | MSC-T |
| T12 | 1 | 2D | 1 | MSC-T |
| T13 | 1 | 2D | 1 | MSC-T |
| T14 | 1 | 2D | 1 | MSC-T |
| T15 | 3 | 2D | 1 | MSC-T |
| T16 | 3 | 2D | 1 | MSC-T |
| T17 | 3 | 2D | 1 | MSC-T |
| T18 | 5 | 2D | 1 | MSC-T |
| T19 | 0 | 2D | 20 | MSC-T |
| T20 | 0 | 2D | 20 | MSC-T |
| T21 | 0 | 2D | 20 | MSC-T |
| T22 | 5 | 2D | 20 | MSC-T |
| T23 | 7 | 2D | 20 | MSC-T |
| T24 | 7 | 2D | 20 | MSC-T |
| T25 | 7 | 2D | 20 | MSC-T |
| T26 | 1 | 2D | 5 | MSC-T |
| T27 | 1 | 2D | 8 | MSC-T |
| T28 | 1 | 2D | 8 | MSC-T |
| T29 | 1 | 2D | 8 | MSC-T |
| T30 | 3 | 2D | 8 | MSC-T |
| T31 | 3 | 2D | 8 | MSC-T |
| T32 | 0 | 2D | 20 | MSC-T |
| T33 | 0 | 2D | 20 | MSC-T |
| T34 | 0 | 2D | 20 | MSC-T |
| I.C1^a^ | 3 | 2D | 20 | MSC-U |
| II.B3^a^ | 3 | 2D | 20 | MSC-U |
| II.B4^a^ | 3 | 2D | 20 | MSC-U |
| II.C1^a^ | 3 | 2D | 20 | MSC-U |

All samples were extracted after culture as described unless otherwise indicated;

a; sample details (culture and extraction) in (Brinkhof et al., 2018)

**Supplemental Table 2. Spearman correlations of *ALCAM* with the other genes tested.**

| Gene | Spearman | |
| --- | --- | --- |
|  | **r** | **p** |
| BSG | -0.05900 | 0.62600 |
| CD59 | 0.36900 | 0.00180 |
| CD63 | 0.17700 | 0.14300 |
| CLIC1 | 0.45500 | 0.00009 |
| CLIC4 | 0.74200 | 0.00000 |
| EDIL3 | 0.72400 | 0.00000 |
| ENG | 0.74300 | 0.00000 |
| EPHA2 | 0.34500 | 0.00359 |
| FN1 | -0.12600 | 0.29800 |
| IGFBP7 | 0.55800 | 0.00000 |
| ITGA1 | -0.03732 | 0.75800 |
| LAMP1 | 0.28000 | 0.01937 |
| LRRC59 | 0.48000 | 0.00003 |
| MCAM | -0.40500 | 0.00061 |
| NECTIN2 | 0.31200 | 0.00874 |
| NT5E | 0.21100 | 0.07900 |
| TFRC | 0.45200 | 0.00010 |
| THY1 | -0.29500 | 0.01344 |
| TLN1 | 0.46400 | 0.00006 |
| TMEM47 | 0.73700 | 0.00000 |
